# “Like a Clinical Nurse Consultant in Your Pocket”—Parents’ and Health Care Providers’ Perceptions of a Co-Designed Smartphone App Following Treatment for Pediatric Cancer: Mixed Methods Evaluation Study

**DOI:** 10.2196/90177

**Published:** 2026-09-07

**Authors:** Hannah Pring, Cinzia R De Luca, Georgia Taylor, Chris Williams, Megan Nagarajah, David D Eisenstat, Maria C McCarthy

**Affiliations:** 1 Paediatric Integrated Cancer Service, The Royal Children's Hospital, South Building, 50 Flemington Road, Melbourne, Victoria, 3052, Australia; 2 Clinical Sciences, Murdoch Children's Institute, The Royal Children's Hospital, 50 Flemington Road, Melbourne, Victoria, 3052, Australia, 61 418 537 742; 3 Children's Cancer Centre, The Royal Children's Hospital, Melbourne, Victoria, Australia; 4 Department of Paediatrics, The University of Melbourne, Melbourne, Victoria, Australia; 5 Innovation and Commercialisation, Murdoch Children's Research Institute, Melbourne, Victoria, Australia; 6 Stem Cell Medicine, Murdoch Children's Research Institute, Melbourne, Victoria, Australia

**Keywords:** digital health, childhood cancer, survivorship, co-design, pediatric, app

## Abstract

**Background:**

Pediatric cancer presents many challenges for young patients and families that extend beyond active treatment. Families report significant unmet information and supportive care needs at the end of treatment (EOT). Digital health solutions, such as patient apps, offer a potential solution to address current gaps in care.

**Objective:**

This study aimed to develop and test a smartphone app called LotusLab, designed to improve access to survivorship information for parents of childhood cancer survivors aged 0 to 18 years, and for young people (aged 12 years and older) at the EOT. In addition, this study aimed to examine parents’ and health care providers’ (HCPs) perceptions of LotusLab during β-testing to inform further app development and refinement.

**Methods:**

Participants were invited to explore the content and key functionality of LotusLab, including an embedded patient-reported outcome measure (PROM). Evaluation of the usability, acceptability, usefulness, and feasibility of the app was conducted through quantitative surveys and qualitative semistructured interviews.

**Results:**

Participants comprised 8 parents whose children had completed cancer treatment, and 9 HCPs. All participants (100%) “agreed” or “strongly agreed” that the app was acceptable; however, HCPs reported more neutral acceptability responses compared with parents. Parents scored app usability more highly, with a mean System Usability Scale (SUS) score of 86.88 (SD 8.74), than HCPs’ mean SUS score of 66.39 (SD 10.16). A common perceived benefit of LotusLab was the provision of trusted information and psychosocial resources. Suggestions for improvement included adding a search function, enhancing the layout, and adding additional topics. All parents completed the app-embedded PROM. Both cohorts noted the value of the PROM in enhancing EOT consultations, while highlighting considerations for the effective integration of and response to PROM data.

**Conclusions:**

Initial data indicate that LotusLab is easy for parents to use and provides value through the provision of EOT information and the opportunity to enhance the use of the PROM. Consistent with the co-design methodology, these findings are guiding the ongoing development and implementation of LotusLab.

## Introduction

A cancer diagnosis in childhood presents extraordinary challenges for patients and families who must navigate complex treatments and profound impacts on family life. The end-of-treatment (EOT) period represents a critical transition period for families, as they re-establish regular family routines while managing treatment side effects and fear of relapse. Multiple studies highlight that this period is marked by significant unmet information and supportive care needs for families [1-4]. Provision of relevant and timely information at the EOT represents a critical strategy for bridging identified gaps in care and mitigating associated psychological distress [5,6]. However, families continue to report barriers to accessing information when needed [2,7-9]. Research examining the optimal delivery of early survivorship information indicates that such information should be tailored to the needs and preferences of the individual, presented in multimodal formats (eg, short videos, animations, and testimonials), and available electronically [10-12].

Mobile apps have been found to be useful and acceptable support tools for young people and caregivers experiencing cancer [13-21]. The potential benefits of apps include increased accessibility to trusted information due to high levels of mobile phone use and opportunities to actively engage users in their health care, including through the delivery of personalized care plans and patient-reported outcome measures (PROMs) [19,21-26]. Notably, there are examples of co-designed digital PROM tools in pediatric oncology that have demonstrated strong and sustained user engagement, particularly during active treatment, providing symptom monitoring and delivery of tailored information [21,27].

Although there are positive examples of progress in the development of apps and digital PROMs, real-world uptake and ongoing engagement with digital health tools remain low, often due to a lack of robust co-design and testing [28,29]. Therefore, to ensure ongoing sustainability, it is essential to actively engage users in all phases of the development and testing of mobile interventions [24,30].

Despite an increasing number of apps developed for families experiencing a cancer diagnosis [23], none were identified that provide information for both young people and parents, and specifically target the EOT transition within a pediatric setting. This paper reports the findings from a study undertaken during the β-testing phase of the co-design of a digital survivorship companion app called LotusLab. The aim of this study was to examine parent caregivers’ and health care providers’ (HCPs) perceptions of the app, including early-phase testing and perspectives on the use of a proxy-reported outcome measure embedded within the app.

## Methods

### Setting

This study was conducted at The Royal Children’s Hospital (RCH), a major tertiary pediatric hospital in Melbourne, Australia.

### Study Design

#### Study Phases

**Figure 1.**
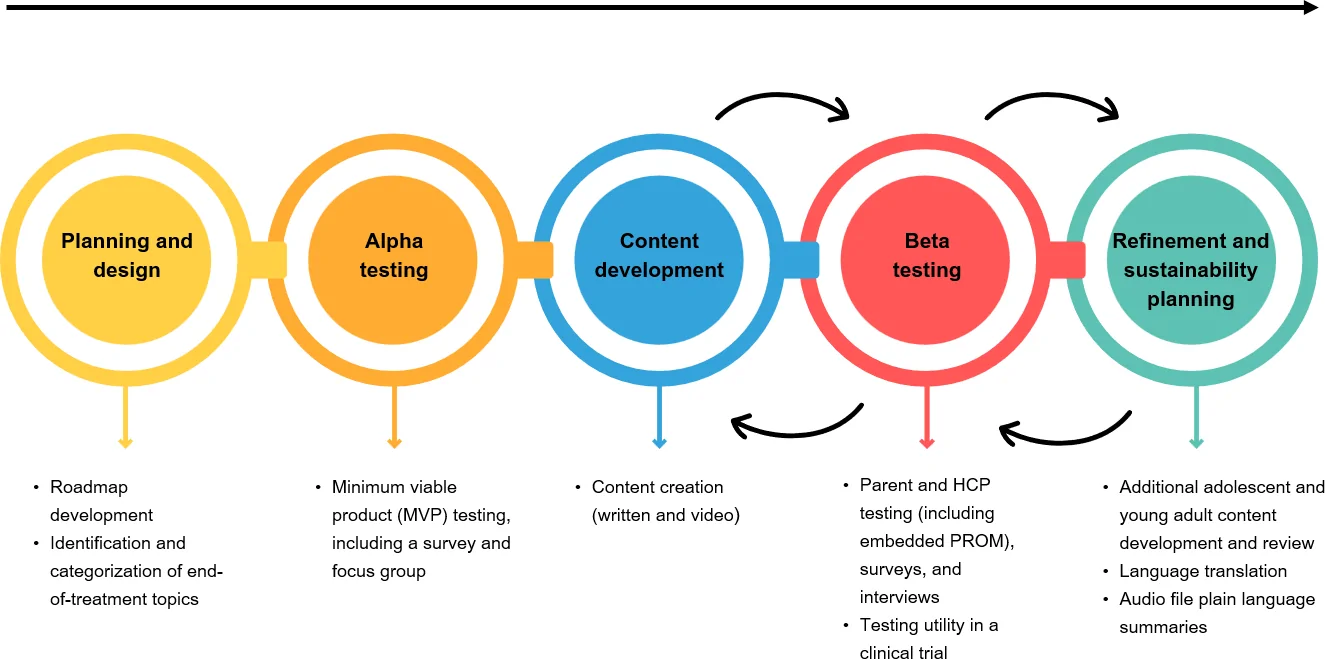
LotusLab co-design and development process. Co-design activities with parents, young people, and HCPs. HCP: health care provider; PROM: patient-reported outcome measure.

Our research team has undertaken a 3-year mixed method study to co-design, develop, and test LotusLab, a digital hub for information after childhood cancer. LotusLab is designed for caregivers of childhood cancer survivors and for young people (aged 12 years and older) at EOT. LotusLab has been co-designed with young people, caregivers, and HCPs in alignment with participatory design principles [31]. Figure 1 shows an overview of the co-design activities across the app development and testing process.

**Figure 2.**
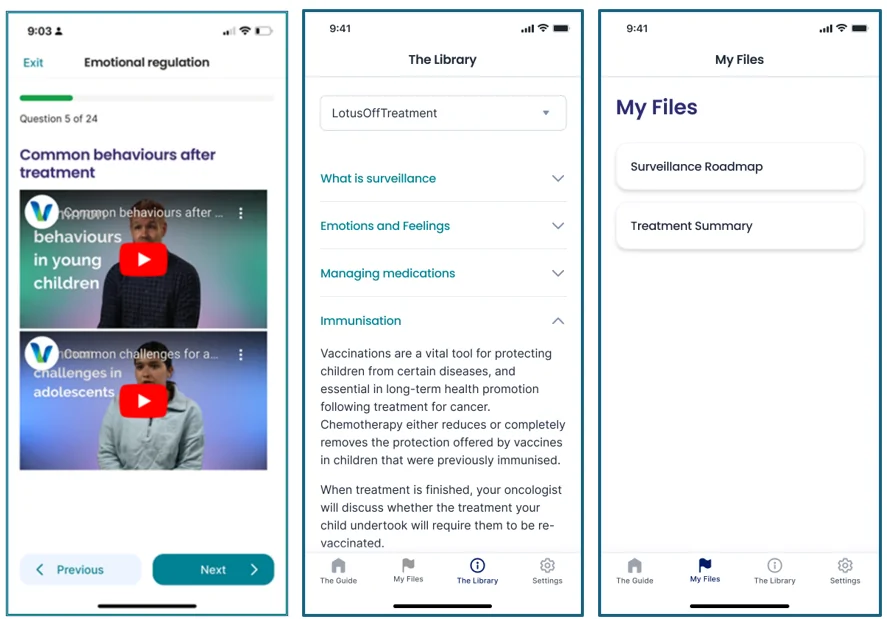
LotusLab screenshots. Both individuals in the screenshots have provided written consent for their images to be used for the purposes of this manuscript.

As a result of this iterative development process, the LotusLab prototype, available for β-testing, provided tailored information and videos, surveys, PROMs, notifications and reminders, and storage of personal survivorship documentation (manuscript under review). Figure 2 shows screenshots of the prototype.

Following planning, design, α-testing (ie, early-phase internal testing), and content development phases, the current study was conducted during the β-testing phase, which focused on testing performance and experience in “real-world” environments. The aim was to evaluate parents’ and HCPs’ perceptions of LotusLab and the use of a proxy-reported outcome measure embedded within the app. Usability, acceptability, usefulness, and feasibility (refer to Table 1 for descriptions) were evaluated through quantitative surveys and qualitative semistructured interviews. Descriptions of these 4 domains were informed by digital health evaluation and implementation science literature [32-34] to ensure that we captured technical outcomes related to the app itself, as well as implementation factors.

**Table 1.** Template analysis theme descriptions.

A priori themes	Description theme inclusions
Theme 1: usability	User-friendliness, ease of use, and efficiency, including navigation, interface, intuitiveness, and ideas for improved functionality
Theme 2: acceptability	Content suitability, relevance, alignment with expectations, and likelihood of recommending. Encompasses all content feedback
Theme 3: usefulness	Ability of the app to provide benefit/value add/impact. Usefulness in meeting needs/requirements. Effectiveness in delivering desired outcomes
Theme 4: feasibility	Practicalities of implementation including timing, barriers, enablers, and ongoing sustainability

#### Participants

Parents or caregivers (hereafter referred to as parents) were eligible to participate if their child (aged 0‐18 y) had finished cancer treatment for any cancer diagnosis within the previous 14 months and was receiving medical cancer surveillance at The RCH’s Children’s Cancer Center (RCH CCC). Eligible clinicians included RCH CCC medical or specialist nursing staff who provided direct clinical care and coordination to families recruited into the study.

#### Procedure

Parent participants were identified by RCH CCC clinical staff, who introduced the study and provided an information letter. A member of the research team contacted the parent within 2 weeks to ascertain their willingness to participate. Medical and specialist nursing staff who were directly involved in the care of the recruited families were approached via email to participate in the study. All participants provided written consent. Following parent and HCP consent, the study team used the hospital appointment booking system to identify and record the date of the patient’s next medical surveillance appointment.

Parents and HCPs were sent instructional emails detailing how to download and explore LotusLab, including how to enroll in the “Lotus Off Treatment” program. HCPs are integral to the successful implementation of digital health interventions. Therefore, as potential deliverers and trusted advocates, HCPs were asked to explore the parent (end user) interface and to evaluate the potential value and use of LotusLab to support their patients and families.

Once enrolled, participants had access to 15 topics, comprising videos and written information and resources tailored to parents and related to the EOT period. If available, the patient’s personalized treatment summary and surveillance roadmap were uploaded to the app. All participants were encouraged to explore the LotusLab layout and content. In addition, parents were asked to complete an in-app survey called “About You and Your Child” to provide basic demographic data and to self-rate their digital literacy. Within the survey, parents provided details about their child’s age, which triggered the in-app release of the corresponding parent proxy-reported Pediatric Quality of Life Inventory (PedsQL; version 4.0), a widely used, validated 23-item measure assessing child health-related quality of life across 4 dimensions: physical, emotional, social, and school functioning [35]. Participants received email and mobile notifications alerting them to the availability of the PedsQL for in-app completion.

Parents were asked to complete the PedsQL within 2 weeks prior to their child’s appointment. Although the PedsQL is validated for self-reporting by children aged 5 to 18 years [35], this early-phase testing was designed to capture parents’ experiences only and therefore included proxy reports for children aged 2 to 4, 5 to 7, 8 to 12, and 13 years and older. Further development and testing of the adolescent and young adult interface are ongoing.

PedsQL results were exported from the app by the study team, and subscale and total scores were manually calculated as per standardized scoring instructions. This procedure was undertaken due to app capability limitations at this phase of testing and the ongoing development of the clinician user interface. Preliminary testing focused on initial perceptions of parents and clinicians regarding the use of and discussion of the PROM in the real-world clinical setting. To support HCP understanding and interpretation of PedsQL domains and scores, and to aid consultation discussions, a custom PedsQL report was shared via email with the relevant HCP prior to the patient’s upcoming appointment (Multimedia Appendix 1). Parents and HCPs were encouraged to discuss the results of the PedsQL during the medical consultation. A retrospective note audit was undertaken to capture any documentation of the PedsQL results and discussion.

Following the consultation, participants were invited to complete usability and acceptability surveys administered via REDCap (REDCap Consortium) software [36] and to take part in an online semistructured interview to discuss their experience with LotusLab.

### Measures

#### Demographics

Brief demographic data related to patient and family factors (eg, diagnosis, age of the child, etc) were captured via an inbuilt survey titled “About You and Your Child.” Additional treatment information or relevant dates were accessed via the electronic medical record (EMR) and stored securely. Information related to HCPs (eg, discipline, role, primary institution, and years of oncology experience) was gathered during the consent process, either verbally or via email.

#### Digital Health Care Literacy Scale

Parents’ digital literacy level was captured to enhance the accuracy of usability testing and to interpret differences between user abilities and issues with the LotusLab design and functionality. The validated 3-item Digital Health Care Literacy Scale was selected to assess participants’ comfort and confidence with mobile technology, specifically the ability to install and use apps and solve technical issues on their own [37].

#### System Usability Scale

Usability was measured and benchmarked with the System Usability Scale (SUS), a validated and widely used measure for assessing the usability of digital products [38,39]. The short 10-item questionnaire shows strong internal consistency and construct validity [40], and the benchmark mean usability score of 68 (SD 12.5) is considered appropriate for assessing the usability of digital health apps across different contexts [39]. Scores greater than 51 can be interpreted as “okay,” scores of 75 and above can be interpreted as “good,” and scores of 86 and above as “excellent” [41].

#### Adapted Abbreviated Acceptability Rating Profile

Acceptability was measured using a study-specific scale that was adapted from the 8-item abbreviated acceptability rating profile (AARP) [42]. Wording of the items was adapted to assess the app (eg, “I would be willing to recommend this app to other parents”), and items were scored on a 5-point Likert scale (strongly disagree, disagree, neutral, agree, and strongly agree; Multimedia Appendix 2).

#### Semistructured Interviews

Semistructured interviews were conducted with parents and HCPs to elicit in-depth perceptions of the app. Interviews were recorded, transcribed, and deidentified for analysis in NVivo (version 15), a software program for data analysis [43] (Multimedia Appendix 3).

### Data Analysis

Descriptive statistics were used to summarize data from the measures outlined above. For the Digital Health Care Literacy Scale, frequencies of responses were calculated. SUS mean scores and score ranges were calculated to provide an overall indicator of usability; frequencies and percentages from the adapted AARP were summarized, describing the proportion of participants who rated the intervention as acceptable.

Template analysis was selected for qualitative analysis of semistructured interviews. Template analysis involves the development of a predetermined coding template that can be adapted in response to the data. It is considered a practical and rapid method well suited to iterative design cycles [44]. In our study, 4 a priori themes were selected to guide analysis and address the study objectives: usability, acceptability, usefulness, and feasibility. Although some additional subthemes were predetermined (eg, barriers and enablers to implementation), others were generated and validated using thematic analysis. To enhance reliability, 2 research team members (HP and MCM) independently coded 3 interviews with parents and HCPs and compared their coding. Consensus on the type of content that related to each a priori theme was reached (see Table 1 for definitions). Remaining transcripts were analyzed by HP with ongoing review by MCM.

### Ethical Considerations

The study was approved by the RCH Human Ethics and Research Committee (#85334). Informed consent was obtained from all participants. All data were securely stored on servers and software approved for the use of protected health information. Additionally, data were deidentified upon collection and stored under a study identification number. Participants did not receive compensation for participation in this study.

## Results

### Study Participants

Eight parents (7 mothers) of children with a range of diagnoses, aged between 3 and 17 years, were approached and consented to participate in the study. All participants (100%) reviewed the app content and provided feedback via online surveys and semistructured interviews.

Twelve HCPs consented to the study; 3 did not respond to follow-up and did not participate in interviews or surveys (75% response rate). Nine HCPs (5 oncologists and 4 advanced practice nurses) reviewed the app content and completed the online usability and acceptability surveys. Eight participated in interviews; 1 HCP was unavailable due to extended leave. Participant characteristics are presented in Table 2.

**Table 2.** Participant characteristics.

Participant characteristics	Value, n
Parent characteristics (n=8)	
Parent/caregiver age (y)	
25‐34	1
35‐44	5
45‐54	2
Parent sex	
Female	7
Male	1
Child’s diagnosis	
Acute lymphoblastic leukemia	3
Brain/CNS^a^ tumor	1
Solid tumors	4
Child’s current age (y)	
0‐5	2
6‐12	2
13‐17	4
Child’s treatment type^b^	
Chemotherapy	8
Immunotherapy	1
Radiation therapy	3
Surgery	6
Region	
Metropolitan	7
Regional	1
Language other than English spoken at home	
No	7
Yes	1
Education level	
High school diploma	2
Bachelor’s degree	3
Graduate certificate	1
Postgraduate degree	2
Phone type	
Android	2
iOS	6
Health care provider characteristics (n=9)	
Role	
Nurse practitioner	2
Consultant oncologist	5
Clinical nurse consultant	2
Age (y)	
35‐44	6
45‐54	2
55‐64	1
Sex	
Female	7
Male	2
Years of oncology experience (y)	
0‐5	1
6‐10	2
11‐15	3
16‐20	1
>20	2
Phone type	
Android	3
iOS	6

a CNS: central nervous system.

b A child can undergo multiple treatment types.

### Parent Results

#### Digital Health Literacy Scale

**Figure 3.**
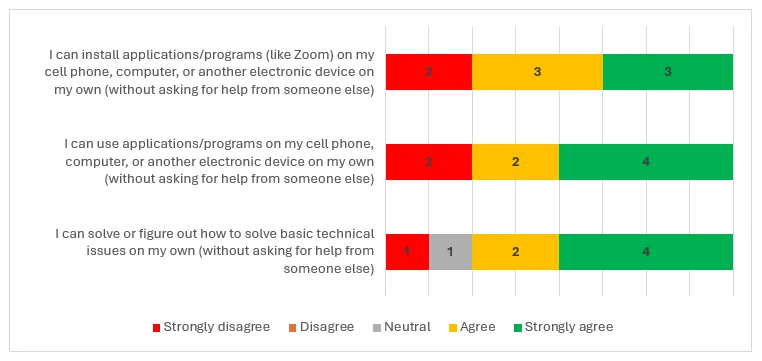
Parent Digital Health Literacy Scale summary.

Figure 3 presents digital literacy results. Six of the 8 parents (75%) “agreed” or “strongly agreed” that they could use or install an app on their device without asking for help and indicated that they could solve basic technical problems on their device. Two (25%) parents “strongly disagreed” that they could do this.

#### Usability and Acceptability of the App

Parents responded with a high mean SUS usability score of 86.88 (SD 8.74; range 75‐100) compared with the benchmark usability score of 68 [39]. All parents “agreed” or “strongly agreed” that the app was “quick to learn” and that they felt confident using the app. Parents individually rated usability as either “excellent” (5/8) or “good” (3/8).

**Figure 4.**
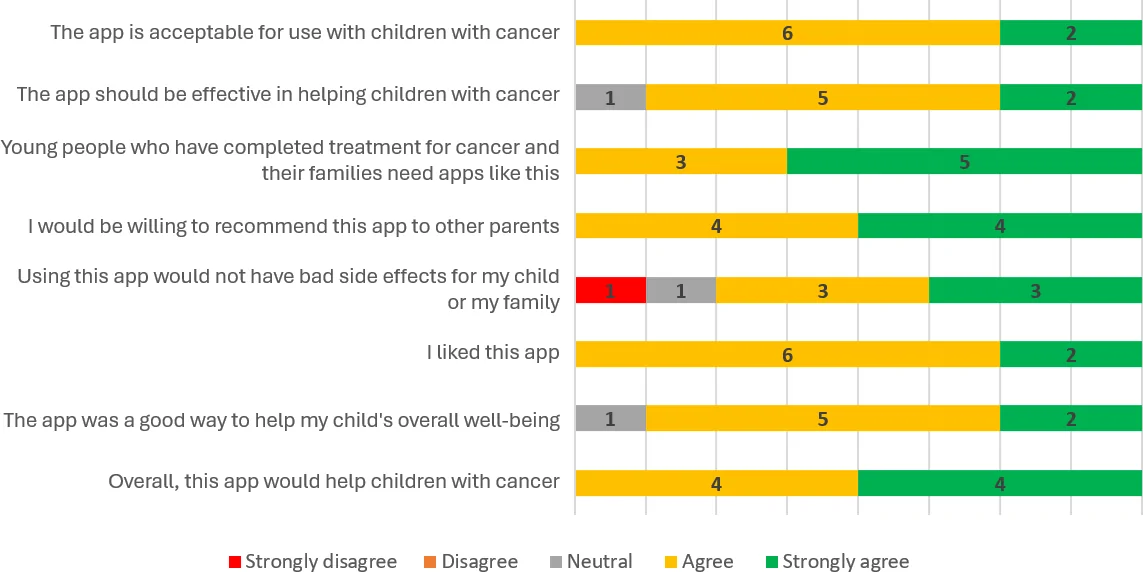
Parents’ ratings of LotusLab acceptability.

All parents “agreed” or “strongly agreed” that the app was fit for purpose and that a digital solution such as LotusLab was needed for young people who had recently completed treatment for cancer. Figure 4 shows parent ratings of LotusLab’s acceptability.

### HCP Results: Usability and Acceptability of the App

**Figure 5.**
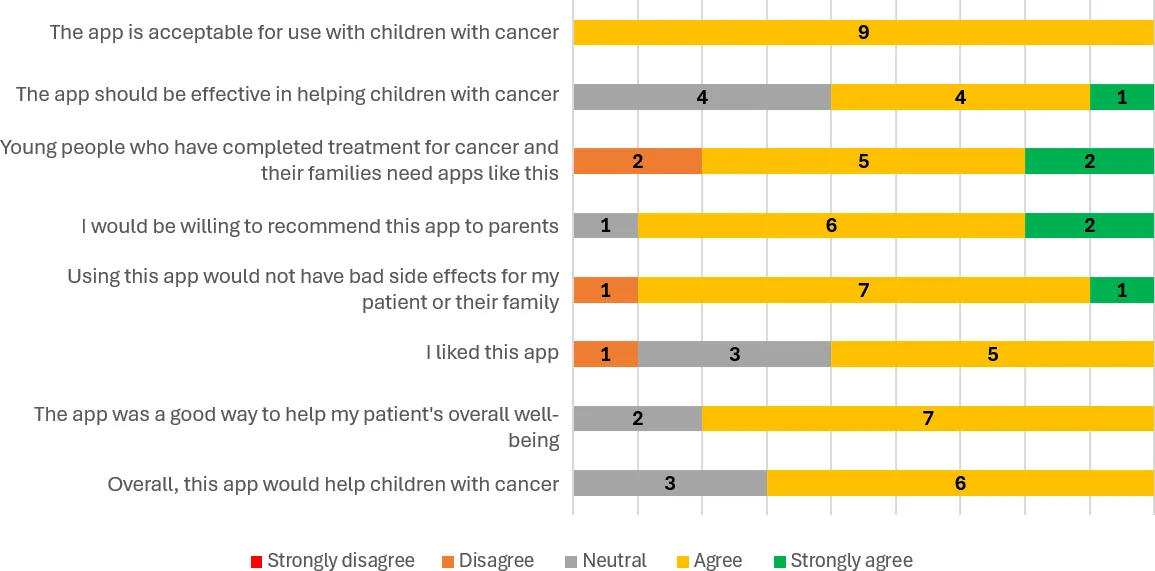
Health care providers’ ratings of LotusLab’s acceptability.

HCPs rated the usability of the parent-facing app in the “okay” range, with an overall mean SUS score of 66.39 (SD 10.16; range 45.0‐77.5). All providers “agreed” that the app was “acceptable for use with children with cancer.” Seven of 9 HCPs (77.8%) “agreed” or “strongly agreed” that young people who had completed treatment and their families need apps like this, and 8 (88.9%) “agreed” or “strongly agreed” that they would be willing to recommend the app to parents. Figure 5 shows HCP ratings of LotusLab acceptability.

### Usage of the App-Embedded PROM (PedsQL)

All 8 parents completed the PedsQL measure embedded within the app. Four participants had a discussion with the oncologist regarding their questionnaire results. One parent stated that, although they did not discuss the questionnaire specifically, they felt the results informed the consultation, as areas of concern were brought up by the consultant and addressed. For 1 parent, the survey was brought up for discussion by the consultant; however, the parent “didn’t bother going into it,” as they felt a different HCP would be better placed to discuss the results. One parent was unsure whether the PedsQL was discussed, as their partner attended the consultation. One parent stated that the PedsQL was not discussed.

A retrospective review of EMR notes found that 4 of 8 clinicians documented the areas of need identified by the PedsQL and the outcomes of the discussion. One clinician documented the parent’s participation in the study but did not directly reference the PedsQL, and 3 clinicians did not reference the PedsQL in the medical record. Parents’ experiences of completing the questionnaire within the app and discussing the PedsQL are captured in the semistructured interviews, as are HCPs’ experiences of receiving and using the PedsQL results. Reporting of PedsQL scores is outside the scope of this study.

### Qualitative Interview Results

#### Interview Characteristics

Semistructured individual interviews were conducted online (using Microsoft Teams) with both parents and HCPs. Interviews ranged from 19 to 41 minutes and were conducted between October 2023 and April 2024. Refer to Table 1 for a priori theme definitions and Table 3 for themes, subthemes, and exemplar quotes from parents and HCPs.

**Table 3.** Exemplar quotes from semistructured interviews with parents and health care providers (HCPs).

Themes and subthemes	Quotes
Theme 1: usability	
Navigation	*“*Usability...was spot-on...it seemed to be...very clear and clean.” (Parent 6) “I liked the idea of all the activities which allowed people to pick and choose the ones that they liked to do*.*” (HCP 10)^a^
Look and feel/design	“*...*whilst aesthetically some people might think it’s a bit basic I actually just found that the basic works so why mess with it?” (Parent 1) “...more navigational like a ‘Choose Your Own Adventure’*...*” (HCP 7)
Functionality	“...I can’t see the completed activities. I think that’s – definitely should be fixed because it would be good to go back and redo some of these or just as a bit of a memory trigger.” (Parent 6) “I do think that search function would be valuable*.*” (HCP 5)
Theme 2: acceptability	
New content suggestions	“Quick links...I’ve got some patients now off therapy a year and they’re asking me about immunisation...on a weekly basis.” (HCP 11)^a^
Content relevancy	“All the videos are really good, especially all the videos that had parents...just made everything so much more relatable and also really calming...” (Parent 3) “...the things that are more of value in the app...you know the emotional development of kids or the emotional responses that they might have after finishing treatment.” (HCP 1)
Content accessibility	“The videos were good perhaps from another language perspective too. Some people find it easier to listen than to read.” (Parent 2) “...obviously the topics are good, but if someone is not as health literate then the text heavy thing actually might be a barrier to them...” (HCP 2)
AYA^b^ acceptability	“...it depends on the kid...if it’s really wordy with modules and things they might feel like it’s homework...” (Parent 4) “...I think there’s some really good key bits but...for a young person I think would look very different.” (HCP 7*)*
Overall experience	“...I was like oh god, I wish I had this app 12 months ago because...I could have just gone to the app and navigated my way through and got the information that I needed.” (Parent 1)
Theme 3: usefulness	
Benefits/value add	“I think that this is like a really nice CNC [Clinical Nurse Consultant] in your pocket...you can just refer back to it.” (Parent 3) “...a nice kind of point of connection still between our service and then coming off treatment...I think, to give them a bit of security...” (HCP 2)
PedsQL^c^ usefulness	“It’s quite meaningful to be asked those kinds of questions...really nice to feel like...someone’s checking in with us.” (Parent 3) “...a really nice kind of segway...I’ve got this survey I can see that sleep, pain and mood are a problem...So I think that using them in a consult...would be super helpful...” (HCP 3) “...it can take up a lot of time and you’re not in the right position to solve a lot of those issues either...” (HCP 6)
Treatment summary and surveillance roadmap	“But if it’s there on an app and you can say look, they said I was to do this or do this...and if you can show them it’s going to make things so much easier. So it would be really good to have access.” (Parent 6) “...he can just go like this and say this is my diagnosis and my treatment. Wouldn’t that empower him? Wouldn’t that have been amazing?” (HCP 11)^a^
Theme 4: feasibility	
Barriers to implementation and ongoing engagement	“I know that my son has been resistant to...Who have I got to talk to now?...I’m ready to move on with the next phase of my life.” (Parent 4) “...like I’ve got loads of apps on my phone...like I turn off a lot of notifications...I think that’s the tricky thing about an app.” (HCP 3)
Enablers to implementation and ongoing engagement	“...I don’t know if she [CNC] has like an orientation checklist...or maybe even like a welcome email from her where maybe she...can just send out the link to this app maybe.” (Parent 3) “I think it’s just the exposure to it...the CNCs who have the relationships with the family...getting it into the Facebook groups and all of those sorts of things, that’s where it’ll all take-off...” (HCP 10)^a^
Timing and introduction	“I know it’s an app for after treatment but for me it was very lonely during treatment...I think if there was a whole aspect of the app where it was what to expect going into it ‘cause it’s so scary and you’ve got no idea.” (Parent 5)

a A total of 12 HCPs were recruited, and 8 participated in interviews; the HCP number aligned with the quote represents the number allocated during sequential recruitment.

b AYA: adolescent and young adult.

c PedsQL: Pediatric Quality of Life Inventory.

#### Theme 1: Usability

Parents felt that LotusLab was easy to navigate and appreciated the “clean” and “clear” look and feel. They valued the ability to choose topics according to personal interest and importance. Practical suggestions for improvement centered on the ability to view completed topics and PROM results once submitted, the ability to share content with others (eg, friends, medical team, and community health), as well as a search facility.

Overall, HCPs also found the app easy to navigate; however, they had more suggestions about the app layout, suggesting topic groupings such as “emotions” and a “more colorful and tiles layout” could help make it easier to navigate. HCPs echoed parents’ comments that the ability to share content and reaccess information through a search function and “Quick Links” sections would enhance the user experience.

#### Theme 2: Acceptability

Parents commented positively on the relevant and relatable content. Two parents stated they wished they had access to the app earlier. There were many suggestions for additional content (eg, development of a frequently asked questions section, information on how parents can support adolescents, and the need for additional videos from the perspectives of children and young people). Specifically, 2 parents reflected on the need for additional content for young people diagnosed with brain tumors, addressing the impacts of cognitive impairment on returning to school and maintaining friendship groups. Parents provided positive feedback about the video content and its ability to enhance accessibility for users with low general and health literacy levels.

HCPs provided positive feedback on the app content, particularly in relation to the videos and psychosocial and emotional needs information at the EOT. There was feedback that additional app content would be needed to enhance its relevance and suitability for patients who had undergone bone marrow transplant and those with brain tumors, due to specific medical and psychosocial needs. HCPs felt that while an app is an appropriate mechanism for young people to access information, LotusLab required enhancements to make it more engaging for young people, such as reducing the amount of text, reordering content to ensure videos always appear at the top, and making it look less like a “learning module.” HCPs suggested that information relating to the LotusLab sign-up process should also be available in languages other than English to ensure equity of access.

#### Theme 3: Usefulness

Parents felt the app provided additional value by collating trusted information in one place (taking out the “legwork”), providing education, alleviating concerns, and allowing people to digest information in their own time.

All parents reported that they found the technical process of completing the PedsQL within the app easy. In addition, most found the process meaningful as a mechanism for “checking in.” Two participants did not discuss the survey results with their oncologists, as they felt that these medical practitioners were not best placed to support their identified needs and that it would have been more beneficial to discuss the results with a different member of the health care team, for example, an occupational therapist. One parent found some of the specific survey questions challenging to answer. They felt some questions were not appropriate to their child’s current situation due to the long-term and evolving cognitive impacts of their diagnosis and treatment. This participant cautioned about the importance of delivering surveys like the PedsQL on a case-by-case basis to ensure relevance to a family’s current situation.

Some participants had difficulty accessing their treatment summaries and roadmaps uploaded to the “My Files” section of the app. Despite this challenge, all parents felt that access to these documents would be useful, especially when visiting other health professionals or when they were unable to access the existing EMR patient portal.

HCPs felt the value of LotusLab centered on its ability to address identified gaps by providing connections to information at the EOT, and they regarded the app as “a good bridge.” In addition, HCPs considered that the app could help normalize experiences. Multiple HCPs felt that LotusLab could potentially reduce the need for parents to consult Google and save HCPs time in answering common questions.

HCPs responded positively to the concept of using the app for PROMs, streamlining the data collection process, and using the results as a mechanism for meaningful discussion of broader quality of life issues and concerns. However, HCPs flagged potential concerns about their capacity to review results captured in the app prior to a consultation, and about the risk of identifying needs but feeling they were “not in the right position to solve a lot of those issues.” They acknowledged that identifying a need that is not addressed introduces risk and could potentially result in a negative experience for patients and caregivers.

HCPs noted that access to personalized survivorship documents, such as a treatment summary, could be empowering, especially for young people who do not always have ownership of these documents. One clinician cited specific examples from their career when access to a treatment summary would have been beneficial for both the treating clinician and the patient, for example, when a childhood cancer survivor presented as an adult to an emergency department but had “no idea” which cancer they had or what treatment they had received for their cancer.

#### Theme 4: Feasibility

The interviews explored in detail the potential barriers to engagement and sustainability of the app. Some parents felt they would be less likely to continue engaging with the app the further off treatment they were, or when feeling “ready to get back to normal” and “not wishing to revisit the past.” Two participants noted that there was a risk the app could feel like “homework,” which could reduce engagement among young people.

Parent-identified enablers to engagement and sustainability included promotion of the app by staff, such as specialist nurses, who know the families well and are best placed to discuss what to expect during the EOT period. Parents noted that embedding the app within existing discussions, such as “EOT reviews,” would be helpful and suggested the app could be advertised through posters in waiting areas.

There were mixed perspectives on the timing of the introduction to LotusLab. Although LotusLab was designed to provide EOT information, many parents felt that access to the app would be beneficial in the months leading up to completion of treatment. One parent felt that introduction to the app should be on a case-by-case basis, as the EOT period can be complex and a specific time point can be hard to determine, but most agreed on the value of knowing what to expect. It was also suggested by several participants that the app could have value during active treatment, lending itself well to “different starts.”

HCPs identified barriers to engagement and sustainability, including the risk that only a self-selecting cohort of people would download and access the app and that those with the greatest unmet information needs might require additional support to engage with the app. One HCP noted that many people turn off their notifications; therefore, ongoing engagement could be challenging. In relation to the collection of PROMs, multiple HCPs raised concerns about the capacity to review survey results prior to consultations due to time constraints, as well as safety concerns centered on ensuring that all results are reviewed and acted upon by an appropriate professional in a timely manner.

HCP-suggested enablers also centered on embedding the implementation of LotusLab within existing roles or services, for example, integrating it into a survivorship clinical nurse coordinator role and including it in “new patient packs.” One HCP suggested that the app could be shared through family networks, such as social media groups. One HCP noted that, in order to streamline LotusLab implementation, it could be helpful to undertake robust change management processes with staff. As per parent feedback, HCPs also suggested creating “a version of this at diagnosis” so that the app could become a longer-term companion across the cancer pathway.

## Discussion

### Principal Findings

This study assessed parents’ and HCPs’ perceptions and experiences of a newly developed app and generated key insights for app refinement, further development, and successful implementation within real-world clinical settings.

Overall, survey and interview data indicated that LotusLab is easy to use and acceptable, and is particularly valuable for the provision of survivorship information. Exploration of usefulness and feasibility highlighted the potential benefits of using LotusLab in bridging care gaps and building engagement during the EOT period. Study participants recommended practical suggestions to enhance the user experience and encourage ongoing engagement.

Throughout the development of LotusLab, parent perspectives as end users have been central; however, effective co-design incorporates input from all stakeholders [45]. In this study, we found similarities and differences in feedback between parents and HCPs. Both cohorts found the app easy to use, even for 2 parents who self-rated their digital literacy as low, and both cohorts suggested similar enhancements to improve usability: primarily a search function, additional content for specific cohorts, and quick links. Interestingly, the HCPs scored overall app usability more variably and acceptability lower than the parents, and 1 clinician did not like LotusLab, providing feedback that they personally did not like using apps in general. HCPs provided more feedback on the design and layout of LotusLab, whereas parent feedback focused heavily on the app content. These findings could reflect the fact that the interface and content were designed with and for parents and not HCPs. Higher parent scores could also reflect the significant unmet information needs experienced by families at the EOT [2,3,8], resulting in parents prioritizing access to information over appearance. This notion of significant unmet needs was further supported by several parents who stated they wished LotusLab had been available to them earlier in the care delivery pathway.

PROMs and proxy-reported outcome measures are increasingly identified as critical components of pediatric cancer care. During this early-phase testing, our aim was to explore the potential benefits of developing a PROM interface and how this could be used to support families at the EOT. Although both parents and HCPs placed significant value on being able to complete the PedsQL within LotusLab, both raised concerns about the ability of HCPs to review and adequately act on identified needs. These findings are echoed in other studies exploring the use of PROMs in pediatric oncology settings, identifying challenges with HCP time pressures, lack of workflow integration, and concerns over the inability to translate results into tangible actions and improved outcomes for families [46-48]. Although clinicians found the provision of meaningful summaries helpful in guiding consultations, this approach did present an additional administrative burden for other team members. Although not specific to the EOT, to address these challenges, apps such as Oncology Hub [21] have opted to deliver algorithm-driven tailored information directly to patients, based on PROM scores at the point of completion and time of need.

From a local implementation perspective, further exploration of the LotusLab algorithm’s capabilities and efficient information exchange is warranted. Our findings highlight that health service capacity and approaches to sharing identified issues and concerns are key considerations for the safe, sustainable, and best-practice management of PROMs through apps such as LotusLab, particularly during the EOT and survivorship phases.

Overall, positive findings relating to LotusLab’s usability and acceptability align with other studies of mobile apps for cancer survivors [14,49]. There was agreement that the most significant value of LotusLab was its provision of practical and psychosocial information at the EOT. Parents and HCPs felt the app had the potential to reduce the burden on young people, parents, and clinicians by providing easy access to trusted information during a time associated with significant uncertainty.

Both cohorts identified barriers to long-term app engagement in alignment with a systematic review of smartphone applications for cancer survivors [14], for example, the risk of diminishing engagement over time. This study suggests that the unique feature of inclusion of a personalized treatment summary and roadmap within the app could encourage repeat use and empower young people to actively manage their future health care when no longer in the pediatric setting. Other barriers included users turning off key app features (eg, notifications) and treatment side effects (eg, cognitive impacts) affecting users’ ability to engage with the app [14].

The impact of digital health apps on patient and family survivorship outcomes is poorly understood, often attributed to a lack of relevant randomized controlled trials [14,50]. In addition, many apps in health care settings do not progress past the initial pilot phase. This is often due to limited, short-term funding that affects the capacity to collect long-term outcomes and cost-effectiveness data, preventing the demonstration of value for both health services and families [14,51-53]. As part of our co-design and evaluation process, we aimed to proactively identify and mitigate potential barriers to sustainability and maximize future impact. A larger “real-world” implementation phase is required to fully quantify the value and impact of LotusLab. Implementing LotusLab within existing systems and workflows, as suggested by participants and similar studies [29], and embedding the app within the standard of care could provide an avenue for sustainable operational costs and increased app success. In addition, LotusLab has the potential to support families across the treatment trajectory (not just EOT), thereby providing a user-friendly way to collect important long-term outcomes data and build the case to support ongoing delivery.

### Limitations

This is a small, single-institution study limited to parents and HCPs. Adolescent and young adult (AYA) perceptions were not evaluated during this specific phase. Engagement with young people to develop age-appropriate content and tailor the app to meet their specific needs is currently being undertaken. In addition, a proxy-reported tool was used in this study; therefore, exploration of AYA perspectives on the use of LotusLab for the collection of PROMs is warranted.

Although some existing content is relevant to all families, further stakeholder engagement would be required for implementation in other health service settings and to increase relevance to specific tumor streams and diagnoses. While there are advantages in using generic quality of life measures like the PedsQL, these tools may lack sensitivity related to disease-specific symptoms [26], a finding corroborated by one parent in this study. This finding corresponds to the broader challenge of developing one digital health intervention for a heterogeneous population of childhood cancer survivors and highlights the significance of including tailored measures and content to provide value and improve the user experience.

All study participants were English-speaking; therefore, feedback is not generalizable to families from culturally and linguistically diverse backgrounds. To support the broader LotusLab project goal to increase equitable access to information at the EOT, the translation of app content into other languages is a key component of “Phase 5: Refinement and Sustainability” (Figure 1). Phase 5 also includes the development and testing of audio summaries in languages other than English.

Although the collection and sharing of outcomes data from the PedsQL were considered valuable, further work is required to build LotusLab’s capability to effectively house and address PROM results. At this stage of β-testing, the app had technical limitations related to automated scoring and the generation of clinically meaningful reports. At a systems level, there are known limitations that affect the collection, sharing, and clinical use of electronic PROMs, such as poor platform interoperability and difficulty integrating PROMs into the EMR [48]. We will continue exploring the optimal approach to using LotusLab to support the integration of PROMs in the pediatric EOT setting.

### Conclusions

Initial β-testing data indicate that LotusLab is easy to use, acceptable, and valuable for parents. Parents and HCPs responded positively to using the app to provide trusted information and contributed actionable insights and practical feedback related to enhancements, PROM use, and app implementation and sustainability. Next steps include app refinement, ongoing engagement with AYA, expansion of content development, integration within real-world EOT models of care, and exploration of effectiveness and outcomes.

## Supplementary material

10.2196/90177 Multimedia Appendix 1 Custom Pediatric Quality of Life Inventory (PedsQL) template.

10.2196/90177 Multimedia Appendix 2 Adapted acceptability survey for parents and health care providers.

10.2196/90177 Multimedia Appendix 3 Interview guide for parents and health care providers.

## Abbreviations

AARP: Abbreviated Acceptability Rating Profile
AYA: adolescent and young adult
CCC: Children’s Cancer Centre
EMR: electronic medical record
EOT: end-of-treatment
HCP: health care provider
PedsQL: Pediatric Quality of Life Inventory
PROM: patient-reported outcome measure
RCH: The Royal Children’s Hospital
SUS: System Usability Scale
