# Supplementary material for: “Like a Clinical Nurse Consultant in Your Pocket”—Parents’ and Health Care Providers’ Perceptions of a Co-Designed Smartphone App Following Treatment for Pediatric Cancer: Mixed Methods Evaluation Study
Source: JMIR Cancer. 2026 Sep 7;12:e90177. doi: 10.2196/90177 (PMC13549700; doi:10.2196/90177)
Supplement: Supplementary file 1 [file cancer-v12-e90177-s002.pdf]

Adapted Abbreviated Acceptability Rating Profile (AARP) for Parents

Directions: For each statement below, please fill the circle that best describes your opinion about the use of the LotusLab app in clinical care for children who have completed cancer treatment and their families.

	**Strongly Disagree**	**Disagree**	**Neutral**	**Agree**	**Strongly Agree**
1. The app is acceptable for use with children with cancer					
2. The app should be effective in helping children with cancer					
3. Young people who have completed treatment for cancer and their families need apps like these					
4. I would be willing to recommend this app to other parents					
5. Using this app would not have bad side effects for my child or my family					
6. I liked this app					
7. The app was a good way to help my child's overall well-being					
8. Overall, this app would help children with cancer					

Adapted Abbreviated Acceptability Rating Profile (AARP) for HCPs

Directions: For each statement below, please fill the circle that best describes your opinion about the use of the LotusLab app in clinical care for children who have completed cancer treatment and their families.

	**Strongly Disagree**	**Disagree**	**Neutral**	**Agree**	**Strongly Agree**
1. The app is acceptable for use with children with cancer					
2. The app should be effective in helping children with cancer					
3. Young people who have completed treatment for cancer and their families need apps like this					
4. I would be willing to recommend this app to parents					
5. Using this app would not have bad side effects for my patient and their family					
6. I liked this app					
7. The app was a good way to help my patient's overall well-being					
8. Overall, this app would help children with cancer					
