# Supplementary material for: “Like a Clinical Nurse Consultant in Your Pocket”—Parents’ and Health Care Providers’ Perceptions of a Co-Designed Smartphone App Following Treatment for Pediatric Cancer: Mixed Methods Evaluation Study
Source: JMIR Cancer. 2026 Sep 7;12:e90177. doi: 10.2196/90177 (PMC13549700; doi:10.2196/90177)
Supplement: Supplementary file 2 [file cancer-v12-e90177-s003.pdf]

<u>Interview Schedule: Parents</u>

<u>Introduction: Study purpose and context</u>
Thank you for participating in this study and for your evaluation of the LotusLab app prototype. We are interested in hearing a little bit more about your perceptions of the app in terms of its usability and value to families at the end of treatment.

Before we start I would like to remind you that participation in this study is entirely voluntary and you may withdraw your consent or discontinue this interview at any time.

Questions	Prompts
Did you have any trouble downloading the app onto your phone?	Did it work every time you accessed it, or did you have to download it multiple times?<br>Did you find the sign in steps easy to follow?
How did you find the design and layout of information on the app?	Was it easy to find what you wanted?<br>Did the layout make sense to you?<br>Did you find it appealing?<br>Were there any aspects that were difficult to use?<br>Did you get lost trying to find any of the information?
Did you like the look and feel of the app?	What parts did you like?<br>What parts didn't you like?
Overall, can you tell me what you thought about the information provided by the app?<br><br>Who do you think should introduce and when?	What information did you find useful?<br>What was not useful/relevant to you?<br>Was the information easy/difficult to understand?<br>Was the amount of information on each topic sufficient?<br>Were there any topics you would like to see covered?<br>Did you share any of the information with someone else? Family member, friend?<br>Did you receive the same information from another source?<br>Did the graphics and videos support the written information well?
How did you find filling out the QoL survey on the app?	Did you appreciate receiving a notification and/or reminder to complete the QoL survey?<br>Did you feel you had enough time to complete the survey prior to your medical appt?

	Was this discussed in your medical appt? If so was it helpful to discuss this with your child's doctor/nurse?
Were you able to access your personalised treatment summary and surveillance roadmap?	Were the documents available to you in a timely manner?<br>Was the mechanism for accessing them easy?<br>Did you download the documents and if so was this easy to do?
Did you manage to enrol in the clinical trials program?	Would like to have access to clinical trials via the app?<br>Would you prefer to simply sign up via the app or complete everything in the app
If this app was finalised and available to use now, do you think you would want to download it/use it?	Would you recommend the app to other families whose child had finished treatment for childhood cancer?<br>Would you continue to return to the app to re-read/watch information? What would it need to have you to return to use it time and time again?
<u>Ideas for improvement</u><br>Do you have any recommendations that should be included on the app?	Are there any features you'd like to see added?<br>Is there anything we could do to make the app more useful?
<u>Child friendly version</u><br>Did your child look at the app?	Is there anything that you would not want your child to access on the app?<br>Did your child look at the app? Do you think the app will be useful for patients? If so what age group do you think it could be suitable for?<br>When do you think families should be introduced to the app?
<u>Exit Question</u><br>Do you have anything else you would like to share about your experience of the app?	

<u>Wrap up</u>

Thank you for your time today and your participation in the study.
We look forward to sharing the findings of Phase 2 of the study with you once we have completed the analyses.

Phone Interview Schedule: HCP

Introduction: Study purpose and context

Thank you for participating in this study and for your evaluation of the LotusLab app prototype. We are interested in hearing a little bit more about your perceptions of the app in terms of its usability and value as a tool to assist in the transition of young people and their families from active treatment to post-treatment care.

Before we start I would like to remind you that participation in this study is entirely voluntary and you may withdraw your consent or discontinue this interview at any time.

Questions	Prompts
Did you have any trouble downloading the app onto your phone?	Did it work every time you accessed it, or did you have to download it multiple times? Were the sign-up instructions easy to follow?
How did you find the design and layout of information on the app?	Was it easy to find what you wanted?<br>Did the layout make sense to you?<br>Did you find it appealing?<br>Were there any aspects that were difficult to use?<br>Did you get lost trying to find any of the information?
Did you like the look and feel of the app?	What parts did you like?<br>What parts didn't you like?
Overall, can you tell me what you thought about the information provided by the app?	What information did you think patient's will find useful?<br>What was not useful/relevant?<br>Was the information easy/difficult to understand?
Was the QoL information easy to understand?	Were you able to review the QoL information prior to the appt?<br>Was the QoL information provided in a timely manner prior to your patient's medical appt? Would you have preferred it sooner?<br>Was this discussed in your patient's medical appt?<br>If so was it helpful to discuss this the family?<br>Did this information add anything novel to your consultation?<br>Was the format the QoL was delivered in suitable and easy to understand/interpret? Was there any information missing that would have aided your patient consultation?

Did your patient's parent report using the app?	Did they ask you any questions about the app or the information provided?<br>Did they provide any feedback on what they liked the most about the app or the way information was shared?
If this app was finalised and available to use now, do you think you think the app would be useful to families as part of their end-of-treatment care??	Can you see yourself using the app in your practice, and in what way?<br>Could you integrate the app into your workflow and overall approach to post-treatment cancer care?<br>Would you recommend the app to other families whose child had finished treatment for childhood cancer?<br>When do you think families should have access to this?<br>What do you think the app adds in regard to your care of the patient during this end-of-treatment period?
<u>Ideas for improvement</u><br><br>Do you have any recommendations for other information/topics that should be included on the app?	Are there any features you'd like to see added?<br>Is there anything we could do to make the app more useful?
<u>Child friendly version</u><br>Would you like to see a version of the app for children?	Do you think the app will be useful for patients?<br>If so, what age group do you think it could be suitable for?<br>Is there anything that you would not want your patient to access on the app?
<u>Exit Question</u><br>Do you have anything else you would like to share about your experience of the app?	Do you anticipate any challenges or limitations in using the app for post-treatment cancer care? Please describe?<br>How would you summarize your overall experience with the app in supporting post-treatment cancer care from a healthcare professional's perspective?

<u>Wrap up</u>

Thank you for your time today and your participation in the study.

We look forward to sharing the findings of Phase 2 of the study with you once we have completed the analyses.
